# Morphological and molecular identification reveals a high diversity of *Anopheles* species in the forest region of the Cambodia–Laos border

**DOI:** 10.1186/s13071-022-05167-0

**Published:** 2022-03-18

**Authors:** Canglin Zhang, Chunhai Luo, Rui Yang, Yaming Yang, Xiaofang Guo, Yan Deng, Hongning Zhou, Yilong Zhang

**Affiliations:** 1 Yunnan Provincial Key Laboratory of Vector-Borne Diseases Control and Research, Yunnan Provincial Collaborative Innovation Center for Public Health and Disease Prevention and Control, Yunnan Institute of Parasitic Diseases Innovative Team of Key Techniques for Vector Borne Disease Control and Prevention (Developing), Yunnan Institute of Parasitic Diseases, Pu’er, 665099 People’s Republic of China; 2 Department of Tropical Diseases, Faculty of Naval Medicine, Naval Medical University, Shanghai, 200433 China

**Keywords:** *Anopheles*, Molecular identification, Malaria vectors, Cambodia–Laos border

## Abstract

**Background:**

To develop an effective malaria vector intervention method in forested international border regions within the Greater Mekong Subregion (GMS), more in-depth studies should be conducted on local *Anopheles* species composition and bionomic features. There is a paucity of comprehensive surveys of biodiversity integrating morphological and molecular species identification conducted within the border of Laos and Cambodia.

**Methods:**

A total of 2394 adult mosquitoes were trapped in the Cambodia–Laos border region. We first performed morphological identification of *Anopheles* mosquitoes and subsequently performed molecular identification using 412 recombinant DNA–internal transcribed spacer 2 (rDNA-ITS2) and 391 mitochondrial DNA–cytochrome c oxidase subunit 2 (*mtDNA-COII*) sequences. The molecular and morphological identification results were compared, and phylogenetic analysis of rDNA-ITS2 and *mtDNA-COII* was conducted for the sequence divergence among species.

**Results:**

Thirteen distinct species of *Anopheles* were molecularly identified in a 26,415 km^2^ border region in Siem Pang (Cambodia) and Pathoomphone (Laos). According to the comparisons of morphological and molecular identity, the interpretation of local species composition for dominant species in the Cambodia–Laos border (*An. dirus*, *An. maculatus*, *An. philippinensis*, *An. kochi* and *An. sinensis*) achieved the highest accuracy of morphological identification, from 98.37 to 100%. In contrast, the other species which were molecularly identified were less frequently identified correctly (0–58.3%) by morphological methods. The average rDNA-ITS2 and *mtDNA-COII* interspecific divergence was respectively 318 times and 15 times higher than their average intraspecific divergence. The barcoding gap ranged from 0.042 to 0.193 for rDNA-ITS2, and from 0.033 to 0.047 for *mtDNA-COII*.

**Conclusions:**

The Cambodia–Laos border hosts a high diversity of *Anopheles* species. The morphological identification of *Anopheles* species provides higher accuracy for dominant species than for other species. Molecular methods combined with morphological analysis to determine species composition, population dynamics and bionomic characteristics can facilitate a better understanding of the factors driving malaria transmission and the effects of interventions, and can aid in achieving the goal of eliminating malaria.

**Graphical Abstract:**

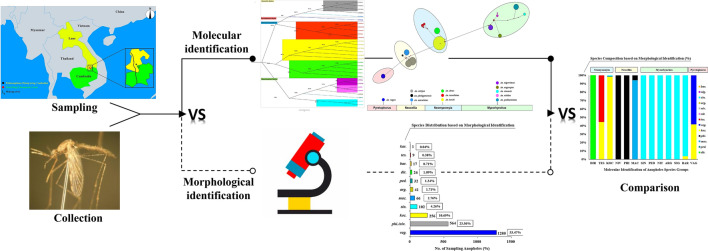

**Supplementary Information:**

The online version contains supplementary material available at 10.1186/s13071-022-05167-0.

## Background

Despite a continued decline in malaria cases (by 74%) and deaths (by 94%) in the Greater Mekong Subregion (GMS) over the past decade, malaria remains a major public health problem [1–3]. Generally, malaria in Southeast Asia is now limited to populations living in the remaining forested regions, mostly in remote areas and adjoining international borders [1–4]. In Cambodia, nearly 61% of the total land area was covered with forest in 2002 [5], of which over 80% was located in malaria-endemic areas [6]. The forest areas are mainly located in the provinces bordering Vietnam, Laos and Thailand. Impacted by the highly efficient forest malaria vectors, people living in villages on the edge of forests or engaged in forest activities are at high risk of malaria [7–10]. Over the past few years, malaria has imposed a major burden on public health in Cambodia, where *Plasmodium vivax* and *Plasmodium falciparum* have been found to coexist [11]. Cases of *P. vivax* are mostly distributed across six northeastern Cambodian provinces, especially in Stung Treng within the Cambodia–Laos border [12]. In Laos, malaria transmission is high in remote, hilly and forested areas, especially in the south [13], where most people are employed in forest-related occupations [14]. Accordingly, malaria is still a serious public health issue in the south [15, 16].

The spread of malaria in the GMS is characterized by vector diversity and great spatial heterogeneity of distribution patterns, and the significance of the respective species in malaria transmission varies widely among different areas [4]. In Cambodia, malaria vectors primarily live in forests close to the borders of Vietnam, Laos and Thailand [17, 18]. *Anopheles dirus*, *An. minimus*, *An. sundaicus* and *An. maculatus* are usually the dominant *Anopheles* species [4, 7, 18], whereas *An. nivipes* and *An. philippinensis* [4] are secondary vectors involved in malaria transmission. In Laos, *An. dirus*, *An. maculatus* and *An. minimus* are recognized as major malaria vectors [7, 13, 18–22]. Other potential vectors (e.g., *An. aconitus*, *An. barbirostris*, *An. nivipes* and *An. philippinensis*) are present [19], although their vectorial capability and ability to transmit *Plasmodium* have been rarely reported.

Correct identification of mosquito species is important to gain a deeper understanding of the composition of mosquitoes in local areas and relevant bionomic features impacting transmission. Morphological identification is the most widely available and generally effective tool at present, but may be complicated by outdated, contradictory and difficult to explain key points [23–25]. Problems with morphological identification (e.g., damage to crucial identifying characteristics, human error, presence of new or cryptic species, species exhibiting overlapping or undocumented characteristics, as well as intraspecific morphological changes) can cause misidentification [25]. Furthermore, to achieve accurate morphological identification, comprehensive and rigorous training is required. Molecular identification can achieve greater support, and it may be more precise in regions of high diversity with considerable numbers of vectors and novel, cryptic and sibling species [23–28].

To gain greater insight into *Anopheles* species diversity and composition in the forested international border region of Cambodia–Laos, molecular methods combined with morphological analysis can play a critical role in characterizing the bionomic characteristics of the *Anopheles* mosquito. Therefore, in this work, morphological identification was first performed, and molecular identification was subsequently conducted using the internal transcribed spacer 2 (ITS2) of recombinant DNA (rDNA) and the cytochrome c oxidase subunit 2 gene (*COII*) of mitochondrial DNA (mtDNA). Furthermore, we compared the molecular and morphological identification, and we conducted phylogenetic analysis of rDNA-ITS2 and *mtDNA-COII* to determine the sequence divergence among species. In addition, we compared rDNA-ITS2 and *mtDNA-COII* with regard to the efficiency of distinction and the genetic divergence among different species, contributing to the molecular identification of mosquitos in malaria vector surveillance. To the best of our knowledge, this was the first comprehensive survey to clarify *Anopheles* species diversity and species composition with molecular identification in the Cambodia–Laos border. In general, we aimed to gain greater insight into the molecular phylogeny of *Anopheles* mosquitoes, in order to enable the formulation of more effective plans for malaria prevention and vector control in the Cambodia–Laos border region.

## Methods

### Site description

**Fig. 1:**
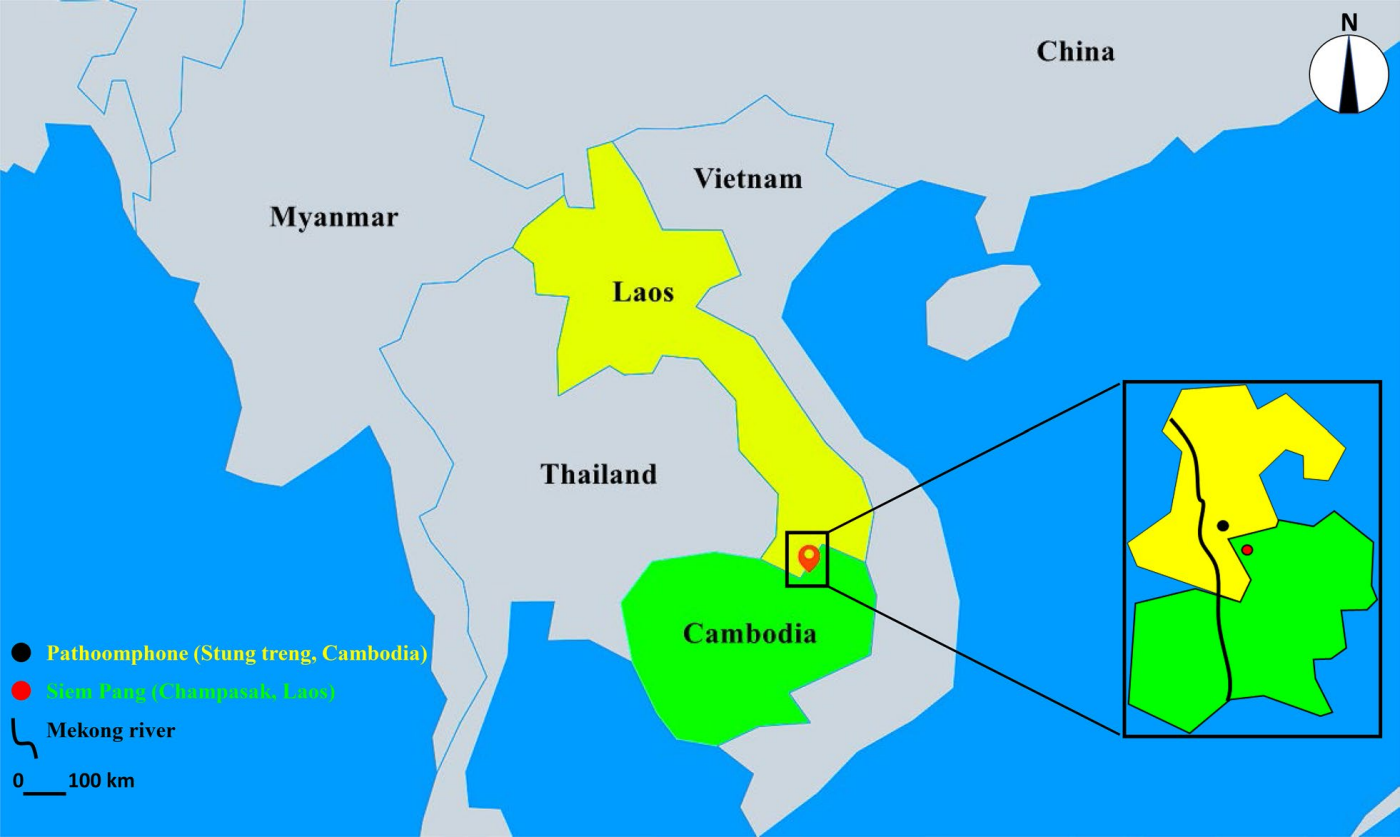
Map of field collection sites in the Cambodia–Laos border. Red circle, Siem Pang County (Stung Treng Province, Cambodia); black circle, Pathoomphone County (Champasak Province, Laos); black line, Mekong River. The shapefile map of Cambodia and Laos was downloaded and prepared using Pixel Map GeneratorBETA online (amCharts, Vilnius, Lithuania) (https://pixelmap.amcharts.com/), which is copyright-free

Siem Pang County (Stung Treng Province, Laos) and Pathoomphone County (Champasak Province, Cambodia) are both located on the east bank of the Mekong River. There is only 59 km between the two sampling sites (Fig. 1), which are both remote, hilly and forested areas along the Cambodia–Laos border. The endemic region occupies an area of nearly 26,415 km^2^ and has about 645,880 residents. The average population density is 24.45 people per square kilometer.

### Mosquito collection and morphological identification

Adult mosquitoes were collected by overnight trapping from 20:00 to 08:00 using battery-operated Centers for Disease Control and Prevention (CDC) light traps (model 1012, John W. Hock Co., USA) hung above the cattle/pig sheds and in human rooms (Table 1). In Siem Pang, CDC light trapping was carried out for four nights (July 20–23, 2018) in cattle and pig pens and six nights (July 24–29, 2018) in human rooms. In Pathoomphone, CDC light trapping was conducted for 15 nights (July 23–August 6, 2017) in cattle or pig pens and six nights (August 1–6, 2017) in human rooms. The live adult mosquitoes were killed by freezing in a refrigerator, and the subsequent isolation and identification processes were carried out according to sex, species and subgroup with the use of a dissecting microscope based on standard keys [29, 30]. All mosquitoes were initially morphologically sorted in the field using the keys reported by Das et al. [31]. Each morphologically identified specimen was kept individually in a 1.5 ml microcentrifuge tube with 75% ethanol and stored at 4 °C for molecular species confirmation and further processing.

**Table 1:** *Anopheles* species composition of mosquitoes trapped by CDC lamps in the Cambodia (KH)–Laos (LA) border region

Trapping sites	Sampling site	No. collected	Vector composition^a^											Sampling time
			*An. philippinensis/nivipes*	*An. maculatus*	*An. vagus*	*An. dirus*	*An. kochi*	*An. sinensis*	*An. argyropus*	*An. peditaeniatus*	*An. barbirostris*	*An. tessellatus*	*An. karwari*	
Cattle/pig pens	Siem Pang (KH)	405	309	49	8	17	21	1	0	0	0	0	0	July 20–23, 2018
	Pathoomphone (LA)^c^	1931	229	2	1269	0	234	98	41	32	16	9	1	July 23–August 6, 2017
Human rooms	Siem Pang (KH)	45	23	15	0	6	0	1	0	0	0	0	0	July 24–29, 2018
	Pathoomphone (LA)^c^	13	3	0	3	3	1	2	0	0	1	0	0	August 1–6, 2017
Total		2394	564	66	1280	26	256	102	41	32	17	9	1	

Trapping sites	Sampling site	No. collected	Vector composition (%)^b^											Sampling time
			*An. philippinensis/nivipes*	*An. maculatus*	*An. vagus*	*An. dirus*	*An. kochi*	*An. sinensis*	*An. argyropus*	*An. peditaeniatus*	*An. barbirostris*	*An. tessellatus*	*An. karwari*	
Cattle/pig pens	Siem Pang (KH)	405	76.30	12.10	1.98	4.20	5.19	0.25	0.00	0.00	0.00	0.00	0.00	July 20–23, 2018
	Pathoomphone (LA)^c^	1931	11.86	0.10	65.72	0.00	12.12	5.08	2.12	1.66	0.83	0.47	0.05	July 23–August 6, 2017
Human rooms	Siem Pang (KH)	45	51.11	33.33	0.00	13.33	0.00	2.22	0.00	0.00	0.00	0.00	0.00	July 24–29, 2018
	Pathoomphone (LA)^c^	13	23.08	0.00	23.08	23.08	7.69	15.38	0.00	0.00	7.69	0.00	0.00	August 1–6, 2017
Total		2394	23.56	2.76	53.47	1.09	10.69	4.26	1.71	1.34	0.71	0.38	0.04	

Adult mosquitoes were collected using overnight trapping with battery-operated CDC light traps, and classified to various species based on morphological identification. ^a^Species composition of mosquitoes described in numbers. ^b^Species composition of mosquitoes described in percentages. ^c^Indicates data collected from our previous studies in Laos [101]

### DNA extraction, ITS2/*COII* amplification and sequencing

Genomic DNA was isolated from individual mosquitoes using the QIAamp^®^ DNA Mini Kit (QIAGEN, Hilden, Germany) following the manufacturer’s instructions. Approximately 674–718 base pairs (bp) of the *COII* gene and 329–717 bp of polymerase chain reaction (PCR) product of the ITS2 region were amplified using primers LEU-F (5′-TCTAATATGGCAGATTAGTGCA-3′) and LYS-R (5′-ACTTGCTTTCAGTCATCTAATG-3′), and ITS2-F (5′-TGTGAACTGCAGGACACAT-3′) and ITS2-R (5′-TATGCTTAAATTCAGGGGGT-3′). *COII* was amplified in a PCR reaction volume of 25 μl with the following cycling parameters: 95 °C, 5 min; 95 °C/1 min, 51 °C/1 min, 72 °C/2 min for 35 cycles; with a final extension at 72 °C for 10 min. ITS2 was amplified in a PCR reaction volume of 25 μl with the following cycling parameters: 94 °C, 2 min; 94 °C/30 s, 50 °C/30 s, 72 °C/40 s for 40 cycles; and a final extension at 72 °C for 10 min. The total PCR reaction volume was 20 μl, and the PCR reagent mixture consisted of 2.5 μl of 10× buffer, 0.2 mM of deoxyribonucleotide triphosphates (dNTPs), 0.3 μM of each primer, 0.05 units of TaKaRa Taq (Dalian, China) and 2 μl of template DNA. The PCR products were analyzed by 1.5% agarose gel electrophoresis stained with GoldView dye (Solarbio, Beijing, China), under ultraviolet transillumination. The sequencing reaction proceeded in both directions using an ABI BigDye Terminator Kit v.3.1 (Applied Biosystems, Thermo Fisher Scientific). Further analysis was conducted with the assistance of an ABI Prism 3500XL Genetic Analyzer (Applied Biosystems, Thermo Fisher Scientific) in Shanghai (Sangon Biotech).

### Molecular processing and sequence analysis

A subset of morphologically identified mosquitoes (*n* = 442 of 2394) were sequenced at the ITS2 region and/or *COII* loci. Samples were first sequenced at the ITS2 locus, and then a subset of samples with successful ITS2 sequences were also sequenced at the *COII* locus.

### Species identification

Molecular identification was conducted blinded to the morphological characteristics to prevent any bias in the analysis. Final species confirmation required high sequence identity (≥ 98%) to voucher sequences in multiple databases [32–65]. ITS2 and *COII* database comparisons of each sample were paired to determine species when ITS2 or *COII* alone did not achieve significant results to the voucher sequence. Consensus sequences were manually checked for the insertion, deletion and repetition regions to ensure that the sequence difference did not expand the divergence or reduce the identity score. Consensus sequences of each sequence group were compared (using BLASTn) to the NCBI Nucleotide database to identify species, and were further compared to the voucher sequences and primers used in diagnostic PCR [49, 57, 66–68] in order to avoid referencing improperly presented or erroneous sequences submitted to GenBank [69, 70].

The keywords “(species name) and ITS2/*COII*” were used to search the ITS2 or *COII* sequences of the 13 *Anopheles* species deposited in GenBank. ITS2 and *COII* sequences which were distant from conspecific sequences after initial sequence alignment were eventually excluded from further analyses.

### Phylogenetic analysis and genetic diversity analysis

Multiple sequence alignment was conducted for both the ITS2 and *COII* sequences in MEGA X [71], and manual adjustments were made using BioEdit 7.0.9 if required [72]. Gaps were excluded from the analysis and characters were unweighted. The phylogenetic trees based on ITS2 and *COII* sequences were both reconstructed using the maximum likelihood (ML) method implemented in MEGA X [71]. Reliability for the internal branch was assessed using the bootstrapping method (1000 bootstrap replicates) [73] to provide a graphical representation of the phylogenetic correlations among different *Anopheles* species. The visualization and editing process for the tree was conducted using FigTree v1.4.2 [74].

Aligned ITS2 and/or *COII* sequences were formatted into a nexus alignment using DnaSP v.5.0 [75]. Nexus formatted sequences were used to create a haplotype network using the median-joining algorithm in Network 4.0 [76]. The final network nodes were colored to reflect species identity. The connection probability threshold of each pair of nodes was set to 0.95.

The intra- and interspecific ITS2/*COII* divergence was measured using the Kimura 2-parameter (K2P) distance model [77] in MEGA X [71].

## Results

### Surveillance of mosquito vectors

**Fig. 2:**
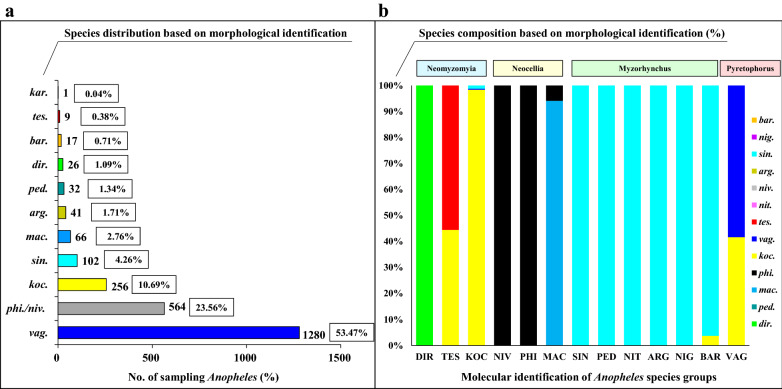
The morphological identification of species based on 2394 *Anopheles* mosquitoes (**a**) and comparison of molecular and morphological identification based on 442 *Anopheles* mosquitoes (**b**). *arg.*: *An. argyropus*; *nig.*: *An. nigerrimus*; *nit.*: *An. nitidus*; *ped.*: *An. peditaeniatus*; *sin.*: *An. sinensis*; *niv.*: *An. nivipes*; *tes.*: *An. tessellatus*; *bar.*: *An. barbirostris*; *dir.*: *An. dirus*; *mac.*: *An. maculatus*; *phi.*: *An. philippinensis*; *koc.*: *An. kochi*; *vag.*: *An. vagus*

A total of 2394 morphologically identified *Anopheles* mosquitoes were collected from cattle/pig sheds or human rooms by overnight trapping along the Cambodia–Laos border (Fig. 1). *Anopheles vagus*, *An. philippinensis/nivipes* and *An. kochi* were the three dominant species and accounted for 53.5% (1280/2394), 23.6% (564/2394) and 10.7% (256/2394) of the total catches, respectively. In addition, *An. maculatus*, *An. dirus*, *An. sinensis*, *An. argyropus*, *An. peditaeniatus*, *An. barbirostris*, *An. tessellatus* and *An. karwari* respectively accounted for 2.8% (66/2394), 1.1% (26/2394), 4.2% (102/2394), 1.7% (41/2394), 1.3% (32/2394), 0.7% (17/2394), 0.3% (9/2394) and 0% (1/2394) (Table 1, Fig. 2a). Compared to cattle/pig sheds, there were quite a few individuals and species of *Anopheles* mosquitoes collected in the human rooms. However, *An. argyropus*, *An. peditaeniatus*, *An. tessellatus* and *An. karwari* were not found in the human room collection (Table 1).

### Molecular species identification

A total of 442 specimens, randomly sampled from all trapping sites and periods, were processed molecularly. Among these, 361 specimens were sequenced for ITS2 and *COII* loci, while the remaining had only one sequence (ITS2 or *COII*). ITS2 sequences representing 412 *Anopheles* mosquitoes were aligned into 12 distinct sequence groups, while *COII* sequences representing 391 *Anopheles* mosquitoes were aligned into 13 distinct sequence groups (Additional file 1: Table S1). Sequence variation in the sequence group was insignificant (less than 2%), i.e., the insertion or deletion did not exceed two bp.

Before a more in-depth database comparison and species-level identification were conducted, different sequence groups were randomly designated as sequence groups 1 to 13. High similarity (≥ 98% identity) to the voucher specimens/sequences in the NCBI Nucleotide database and multiple other databases [32–65], as well as concordant *ITS2-COII* pairs, allowed the preliminary identification of 13 species: *An. dirus* (group 1), *An. peditaeniatus* (group 2), *An. maculatus* (group 3), *An. philippinensis* (group 4), *An. kochi* (group 5), *An. vagus* (group 6), *An. tessellatus* (group 7), *An. nitidus* (group 8), *An. nivipes* (group 9), *An. argyropus* (group 10), *An. sinensis* (group 11), *An. nigerrimus* (group 12), *An. barbirostris* (group 13) (Table 2, Additional file 1: Table S1).

**Table 2:** Molecular identification of species using both ITS2 and *COII* comparisons

Sequence group	No. of samples (ITS2; *COII*)	Sequence length in bp (ITS2; *COII*)	Molecular species ID	Morphology species ID (no.)	No. of sequences identified molecularly	No. of correctly identified specimens based on morphology	% of correctly identified specimens based on morphology
Group 1	22; 21	717; 687	*An. dirus*	d (22)	22	22	100.00
Group 2	18; 18	457; 674	*An. peditaeniatus*	s (18)	18	0	0.00
Group 3	17; 7	329; 685	*An. maculatus*	m (16), phi (1)	17	16	94.12
Group 4	2; 2	353; 685	*An. philippinensis*	phi (2)	2	2	100.00
Group 5	244; 213	400; 718	*An. kochi*	k (242), v (1), s (3)	246	242	98.37
Group 6	11; 12	568; 686	*An. vagus*	v (7), k (5)	12	7	58.33
Group 7	9; 9	467/591; 690	*An. tessellatus*	t (5), k (4)	9	5	55.56
Group 8	4; 3	480; 686	*An. nitidus*	s (4)	4	0	0.00
Group 9	59; 53	356; 690	*An. nivipes*	phi (59)	59	0	0.00
Group 10	8; 8	472; 687	*An. argyropus*	s (8)	8	0	0.00
Group 11	4; 4	469; 686	*An. sinensis*	s (4)	4	4	100.00
Group 12	14; 14	508; 687	*An. nigerrimus*	s (14)	14	0	0.00
Group 13	0; 27	0; 691	*An. barbirostris*	s (26), k (1)	27	0	0.00
Total	412; 391	**–**	**–**	–	442	298	67.42

Morphologically based species identification included: d, *An. dirus*; s, *An. sinensis*; m, *An. maculatus*; phi, *An. philippinensis*; k, *An. kochi*; v, *An. vagus*; t, *An. tessellatus*. Number and percentage of correctly identified morphological specimens are calculated from the number of sequences that were molecularly identified per species

To prevent misidentification, the consensus sequences of each sequence group mapping to the Hyrcanus Group, Maculatus Group, Dirus Complex, Annularis Group and Barbirostris Group were further compared to the voucher sequences and primers applied in PCR diagnostic assays [49, 57, 66–68]. Among them, the species identity of groups 2, 8, 10, 11 and 12 was finally clarified as *An. peditaeniatus*, *An. nitidus*, *An. argyropus*, *An. sinensis* and *An. nigerrimus*, respectively [49]. Comparison of the group 3 ITS2 sequence to specimens applied in the Maculatus Group diagnostic assay [57, 66] demonstrated 99.8% similarity of *An. maculatus*. Group 1 was confirmed as *An. dirus* of the Dirus Complex [67], while group 4 and group 9 were confirmed as *An. philippinensis* and *An. nivipes* of the Annularis Group, respectively [68]. In addition, group 13 was finally clarified as *An. barbirostris* of the Barbirostris Group [78].

### Phylogeny

**Fig. 3:**
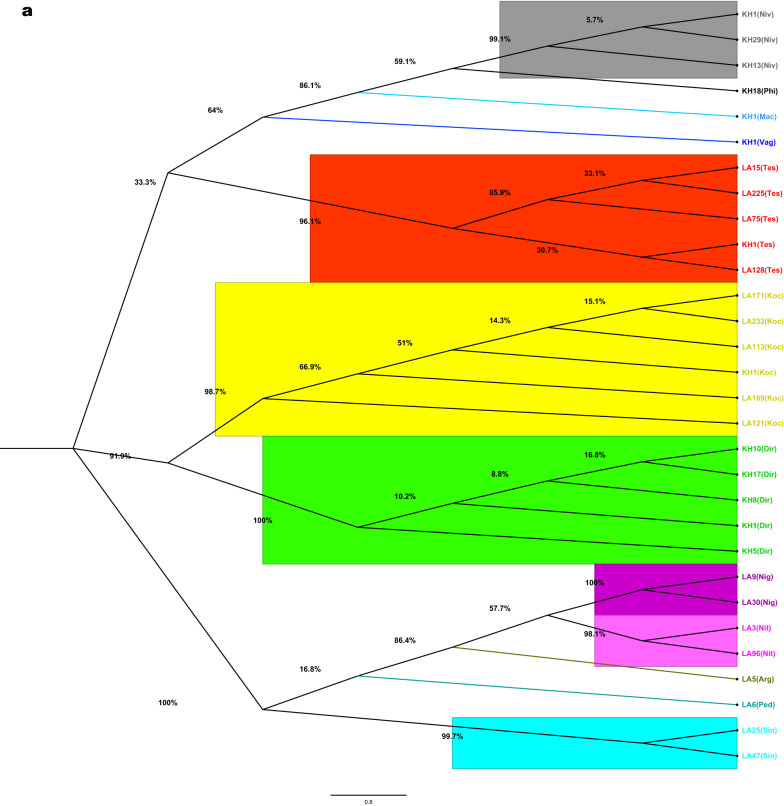

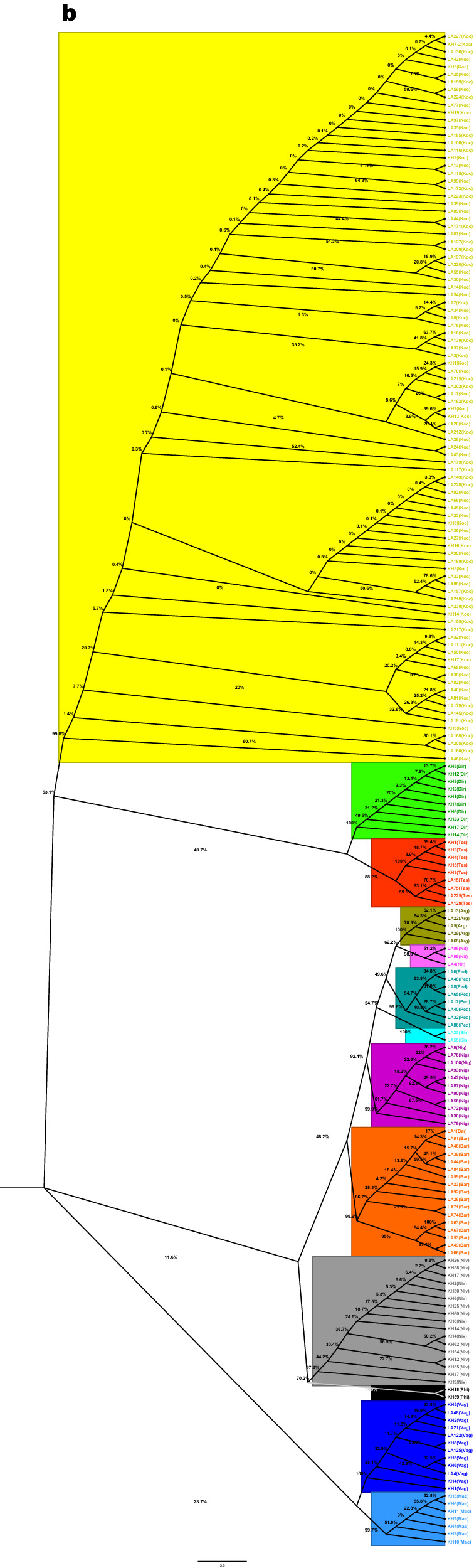
Phylogenetic tree based on 412 ITS2 (**a**) and 391 *COII* (**b**) sequences from this study. Bootstrap values (1000 replicates) of maximum likelihood analyses are shown above/below the main lineages. Lineage designation is indicated on the right. Bars represent 0.8 substitutions per site based on ITS2 and 5.0 substitutions per site based on *COII*

Alignments were first performed on 412 ITS2 and 391 *COII* sequences, and identical sequences from the same data set or species were excluded from the subsequent analysis. Thus, 30 ITS2 and 199 *COII* consensus sequences (haplotypes) were further screened to build a phylogenetic tree (Additional file 2: Table S2). The putative species of the ITS2 or *COII* tree groups were as expected based on their taxonomy (Fig. 3). Based on ITS2 and *COII* sequences, two subgenera (*Anopheles* and *Cellia*) and four series (Myzorhynchus, Neomyzomyia, Pyretophorus and Neocellia) were identified. *Anopheles peditaeniatus* (group 2), *An. nitidus* (group 8), *An. argyropus* (group 10), *An. sinensis* (group 11), *An. nigerrimus* (group 12) and *An. barbirostris* (group 13) were clustered respectively as parts of the Myzorhynchus Series in both trees (*An. barbirostris* group was not present in the ITS2 tree) (Fig. 3). *Anopheles dirus* (group 1), *An. kochi* (group 5) and *An. tessellatus* (group 7) clustered respectively as parts of the Neomyzomyia Series in the *COII* tree (Fig. 3b), while they were separated into two subclusters in the ITS2 tree (Fig. 3a). *Anopheles maculatus* (group 3), *An. philippinensis* (group 4) and *An. nivipes* (group 9) were clustered respectively as parts of the Neocellia Series in the ITS2 tree (Fig. 3a), while they were separated into two subclusters in the *COII* tree (Fig. 3b). *Anopheles vagus* (group 6), a part of the Pyretophorus Series, was clustered in the *An. maculatus* group (group 3) in both trees (Fig. 3).

For a broader comparison, 591 ITS2 sequences and 873 *COII* sequences of 13 *Anopheles* groups were extracted from GenBank, and 88 ITS2 and 308 *COII* sequences (haplotypes) were further screened in total to reconstruct the phylogenetic trees (Additional file 3: Table S3), combined with ITS2 and *COII* sequences (haplotypes) in this paper. After the suspicious fragments were excluded, 118 ITS2 sequences of 12 *Anopheles* groups and 507 *COII* sequences of 13 *Anopheles* groups were conducted to reconstruct the phylogenetic trees. In the new ITS2 and *COII* trees, the four *Anopheles* series were clustered respectively from each other. Furthermore, the *An. barbirostris* group in the *COII* tree was not clustered in the Myzorhynchus Series, and an independent cluster was formed (Additional file 4: Fig. S1, Additional file 5: Fig. S2).

Due to having lower numbers of sequences, closely related species with high sequence similarity, similar distances of different species, and low genetic differentiation within species complex or group, unresolved branches with a bootstrap value under 70–80% or even 50% might exist in building phylogenetic trees, especially in the *COII* trees (Fig. 3, Additional file 5: Fig. S2). Therefore, including more sequences to build a phylogenetic tree or setting a bootstrap cut-off value of 70% or 50% would be a better way to solve these problems.

**Fig. 4:**
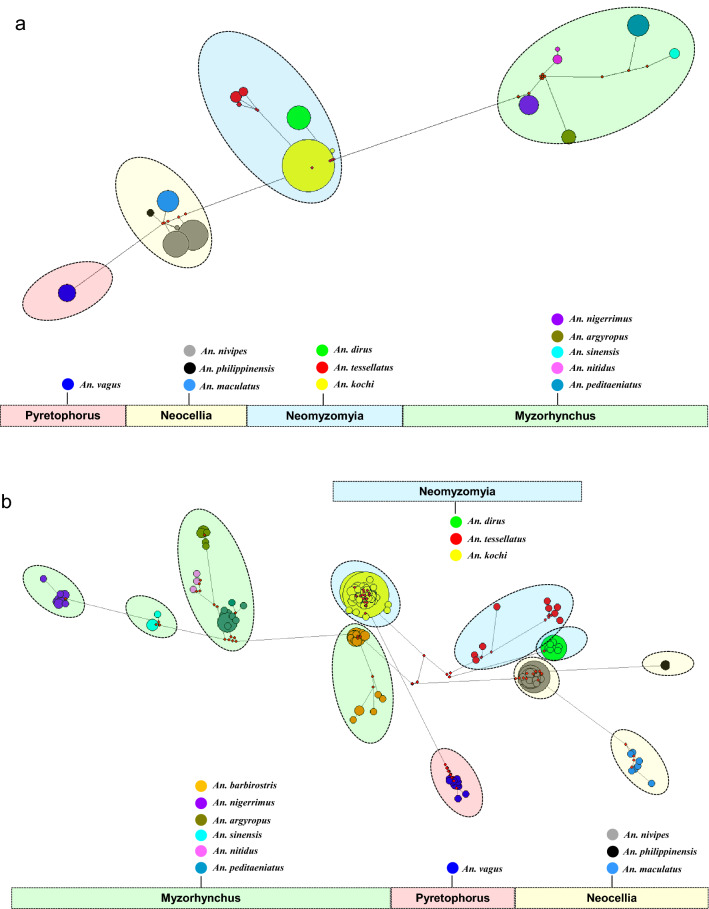
Haplotype network based on 412 ITS2 (**a**) and 391 *COII* (**b**) sequences from this study. Each observed haplotype is indicated by a filled circle, sized according to its frequency and colored according to the *Anopheles* species represented. Haplotype relationships are indicated by lines; mutational steps between haplotypes are represented by the number of lines

The median-joining network based on 412 ITS2 and 391 *COII* sequences in this paper denote the distribution pattern exhibited by haplotype in 13 *Anopheles* groups. There were significant differences in the number of haplotypes and prevalence of individual haplotypes among all the species considered, and considerable divergence was found between main cores of haplotypes and their distinctive species composition in both networks. In the ITS2 network, 18 haplotypes representing 12 *Anopheles* groups fell into four independent series groups, i.e., Myzorhynchus, Neomyzomyia, Pyretophorus and Neocellia (Fig. 4a). Consistent with the ITS2 network, 185 haplotypes representing 13 *Anopheles* groups also fell into four series groups in the *COII* network (Fig. 4b).

### Comparison of molecular and morphological identification

Sequencing demonstrated the presence of 13 distinct sequence groups, while morphology suggested the presence of only seven species. Molecular identification of 13 distinct sequence groups achieved 100% accuracy of all species, whereas very few species were correctly identified morphologically with 100% accuracy or accordance rates. The highest accuracy rates of morphological identification were found in five dominant species from 98.8% to 100%, as *An. dirus* (100%), *An. maculatus* (94.1%), *An. philippinensis* (100%), *An. kochi* (98.4%) and *An. sinensis* (100%), respectively (Table 2, Fig. 2b).

For the other eight distinct sequences molecularly identified to species, the percentage of correctly identified morphological specimens ranged from 0 to 58.3% (Table 2, Fig. 2b). Five *Anopheles* species of the Myzorhynchus Series, i.e., *An. peditaeniatus*, *An. nitidus*, *An. argyropus*, *An. nigerrimus* and *An. barbirostris*, and one *Anopheles* species of the Neocellia Series (*An. nivipes*) were not morphologically identified. Specifically, *An. peditaeniatus*, *An. nitidus*, *An. argyropus* and *An. nigerrimus* were four members of the Hyrcanus Group, *An. nivipes* pertained to the Annularis Group, and *An. barbirostris* pertained to the Barbirostris Group. Due to the misidentification of certain specimens as *An. kochi*, the correctly identified morphological specimens of *An. vagus* (58.3%) and *An. tessellatus* (55.6%) had low percentages (Table 2, Fig. 2b).

### Comparison of resolution of species identification based on ITS2 and *COII*

To compare the resolution of species identification using the two markers and to measure the degree of concordance in the data, we compared the resolution of species identification based on a total of 361 specimens sequenced for both markers. The intraspecific K2P distance of ITS2 reached 0.004 on average, while the interspecific K2P distance varied from 0.193 between *An. nivipes* and *An. philippinensis* to 2.281 between *An. argyropus* and *An. vagus*, with an average of 1.286 (Additional file 6: Tables S4a). The intraspecific K2P distance of *COII* reached 0.007 on average, while the interspecific K2P distance varied from 0.047 between *An. peditaeniatus* and *An. nitidus* to 0.140 between *An. vagus* and *An. nigerrimus*, with an average distance of 0.102 (Additional file 6: Tables S4b). Therefore, the ITS2 and *COII* sequence divergence in intragroup species was approximately 318 and 15 times higher than the average within species, respectively.

In addition, the ITS2 barcoding gap ranged from 0.042 to 0.193, while the *COII* barcoding gap ranged between 0.033 and 0.047 (Additional file 7: Fig. S3). In Additional file 8: Fig. S4, the minimum interspecific divergence is plotted against the maximum intraspecific divergence. It is worth noting that there are more ITS2 than *COII* dots closer to the top left-hand corner of the graph, indicating that ITS2 is a more effective marker than *COII* for species differentiation.

## Discussion

Anopheline vectors in the GMS are incredibly diverse and primarily bite outdoors [9, 17, 79], enabling them to avoid commonly used vector control interventions, including indoor residual sprays or insecticide-treated nets (ITNs). A group of outdoor-biting mosquito species, flexible as to feeding on animals and frequently in humans, is responsible for residual malaria transmission (RMT) in many endemic countries [80], such as *An. dirus* in Southeast Asia [81]. The main vectors of malaria belong to complexes or groups of species that are difficult or impossible to distinguish due to overlapping morphological characteristics [28]. Therefore, understanding temporal vector composition by properly identifying the species along with their bionomic traits may lead to better and more targeted intervention strategies. Recent advances in molecular systematics have provided simple and reliable methods for unambiguous species identification which can achieve greater support and may be more precise in regions of high diversity [23–28].

To achieve the malaria elimination goal for Laos and Cambodia, intervention strategies should meet the requirement of varying and complex transmission dynamics of multiple local mosquito vectors. In this study, we preliminarily characterized the biodiversity of *Anopheles* species in the forested region along the international borders. The diversity of malaria vectors in the Cambodia–Laos border with suboptimal morphological identification highlights the significance of integrating molecular identification into vector studies. The use of molecular methods combined with morphological analysis to determine species composition, population dynamics and bionomic characteristics can aid in determining the drivers of malaria transmission and in intervention effectiveness, as well as in the pursuit of malaria elimination. In this work, molecular identification was conducted based on rDNA-ITS2 and *mtDNA-COII* sequences to identify species with the conservative algorithm outlined above. Moreover, by comparing the molecular and morphological identification and the phylogenetic analysis of both markers to determine the sequence divergence among species, 13 separate species sequences were identified in this border region.

Because of the highly variable morphology and almost identical morphological features possessed by adults of some closely related species [53, 82], accurate distinction between the species within the Hyrcanus Group is difficult when based only on morphological properties, even for trained taxonomists [32, 83]. In this study, a total of 77 individuals were morphologically identified as *An. sinensis*, whereas only four were finally molecularly identified as *An. sinensis*. The remaining 73 identified morphologically as *An. sinensis* were corrected as *An. peditaeniatus* (18/73), *An. kochi* (3/73), *An. nitidus* (4/73), *An. argyropus* (8/73), *An. nigerrimus* (14/73) and *An. barbirostris* (26/73). Likewise, *An. philippinensis* and *An. nivipes* of the Annularis Group showed highly similar morphological characteristics, which might complicate the differentiation of the two species in the adult (especially females) and larval stage [84]. Using adult wing characters, Nagpal and Sharma initially identified the presence of *An. nivipes* from northeastern India [85]. The close resemblance of the two species led to confusion in terms of accurate distributions and yielded inadequate data on relative abundance. In this work, we initially referred to the morphologically identified *An. philippinensis* as *An. philippinensis*/*nivipes* in the vector surveillance (Table 1). However, given the molecular identification, 95.1% (59/62) of the morphologically identified *An. philippinensis* were finally corrected as *An. nivipes*.

*Anopheles vagus* was identified through morphological identification as the dominant *Anopheles* species (53.5%) in southern Laos (Champasak). However, as reported in our previous studies, *An. sinensis* or *An. argyropus* was the dominant *Anopheles* species in northern Laos, including Phongsaly, Luangprabang, Luangnamthat and Odomxay [86–88]. The inconsistency in the major *Anopheles* species between northern and southern Laos may be attributable to differences in sampling season, trapping method or trapping period. It is worth noting that environmental changes caused by human activities or global climate change may affect the spatial distribution or community structure of malaria vectors and malaria transmission dynamics, since malaria vector species have unique niche requirements [89]. However, only 12 *An. vagus* were molecularly identified here, and an in-depth investigation should be undertaken. Moreover, *An. vagus* has been previously suspected of being a species complex [90]. In a study by Davidson, the ITS2 phylogenetic tree indicated that there were two genetically distinct *An. vagus*-like species (AN4 and AN5) [26]. However, we did not identify any genetically distinct *An. vagus*-like species (Additional file 5: Fig. S2). Moreover, the identification of two distinct groups of *An. vagus*, i.e., AN4 (GenBank accession no. MT740902.1) and AN5 (GenBank accession no. MT740903.1), might be due to incorrect determination of their ITS2 region boundaries. AN4 and AN5 ITS2 sequences might be aligned without deleting the partial sequences of 5.8S ribosomal RNA and/or 28S ribosomal RNA.

It is noteworthy that according to the phylogenetic analysis of *An. tessellatus* based on ITS2 and *COII*, two or three subclusters were identified in both trees (Fig. 3, Additional file 4: Fig. S1 and Additional file 5: Fig. S2), which demonstrated that *An. tessellatus* collected in this study should be considered a different subspecies in the Tessellatus Group [91]. Accordingly, the phylogenetic trees were reconstructed based only on the *An. tessellatus* ITS2/*COII* sequences in combination with our original sequences and sequences retrieved from NCBI (Additional file 9: Fig. S5). Interestingly, the present work found that *An. tessellatus* collected from Siem Pang formed a distinct cluster in both trees that was distant from Pathoomphone samples and other geographical samples. The subspecies composition in the Tessellatus Group in this border area should be further investigated.

Furthermore, transmission by “secondary” vectors exhibiting outdoor or early biting behavior might become more important than transmission by primary vectors under high coverage of ITNs [92]. Moreover, secondary vectors might be more effective vectors of *P. vivax* than *P. falciparum*, since the extrinsic incubation period of *P. vivax* is shorter [93]. In northeast Indonesia, *An. kochi* has a habit of biting humans and domestic animals. Peak biting of humans and cattle occurs in the first half of the night [94], with a preference for one or the other depending on the distance to the blood source and its protected conditions. When livestock pens are distributed around human houses, the probability of mosquitoes feeding on human blood is elevated, so the probability of *Plasmodium* sporozoite infection increases [94]. *Anopheles kochi* plays a significant role in *P. vivax* and *P. falciparum* malaria transmission, which has been observed on the Bangladeshi–Indian border [95–98]. It also acts as a potential vector of human malarial parasites in Thailand, with susceptibility to *P. falciparum* and *P. vivax* [99]. In the northern Maluku Islands, a *P. vivax* infection rate of 1.8% (6/336) was found in *An. kochi* samples [100]. In this work, *An. kochi* was one of the dominant species, accounting for 10.7% (256/2394) in accordance with morphological identification (Fig. 2). Our previous studies in Pathoomphone revealed that *P. vivax* sporozoites were detected in *An. kochi* and *An. sinensis*, and the positivity rate reached 2.6% and 2.0%, respectively [101], while no sporozoites of *P. falciparum* were detected in the two species. According to existing research in southern [21] and northern Laos, central Vietnam and northern Cambodia [102], and Kachin State of Myanmar and Yingjiang in China [103, 104], *An. minimus* and *An. dirus* can carry *Plasmodium* sporozoites, whereas neither *An. kochi* nor *An. sinensis* was reported to carry sporozoites. Since *An. sinensis* is experimentally susceptible to *P. vivax*, indicating a potential role as a malaria vector [105], further analysis of *An. sinensis* field samples might reflect the actual status of *Plasmodium* sporozoite-carrying mosquitoes. In-depth research should be conducted to describe the relationship between the bionomic features of *An. kochi*/*An. sinensis* and local malaria epidemics. Likewise, *An. nivipes* was a second dominant species and accounted for 23.6% (564/2394) of the total catches in accordance with morphological identification (Fig. 2). In Cambodia, *An. nivipes* and *An. philippinensis* were found to be secondary vectors in transmitting malaria [4]. *Anopheles nivipes* accounted for 23.5% and 35.7%, respectively, in Preah Vihear and Ratanakiri in northern Cambodia [106]. In Laos, *An. nivipes* was suspected to be one of the dominant species and accounted for 11.6% in central Laos (Khammouane) [107], whereas it constituted over 65% in the southeastern part of Laos (Nongceng) [108].

In addition, mtDNA is suggested to be more effective in determining the possibility of ancient hybridization in mosquito molecular phylogeny, while rDNA has shown higher reliability than mtDNA in resolving the evolutionary issues using the recently diverged taxa or cryptic species of mosquitoes [36] and in establishing species boundaries if they fail to be resolved using mtDNA. The comparison of intra- and interspecific ITS2/*COII* variation in the present study revealed that ITS2 may be a more effective marker for differentiating species than *COII*, which is consistent with previous findings that an effective DNA marker should have a small intraspecific distance and a large interspecific distance [109]. The major downside of using *COII* for phylogenetic analysis is that *COII* may be unable to distinguish between closely related species [110]. Thus, additional research in the Cambodia–Laos border using nuclear and mtDNA sequencing is necessary to accurately identify species.

The discrepancy between morphological and molecular identification highlights the significance of incorporating molecular tools for more effectively distinguishing vector species, especially in areas of high vector diversity. Morphological identification showed the highest accuracy or accordance with molecular identification for the most abundant species groups (e.g., *An. kochi*, *An. dirus* and *An. maculatus*) in the Cambodia–Laos border. However, when less common species were examined, a comparison of molecular- and morphological-based species identity demonstrated inconsistency based upon morphological identification. Finally, all molecularly identified species were mistaken for multiple species when morphological identification was conducted independently. Misidentification caused by morphological identification may have negative downstream effects on the determination of species’ bionomic features, associations of vector status, entomological inoculation rates and impacts on control [111].

## Conclusion

This paper highlights the significance of cross-referencing morphological identification with molecular identification for determining mosquito species composition. Thirteen distinct sequences were identified to species. This is the first study to characterize species composition in the forested international border region of Cambodia–Laos with molecular identification techniques. Future studies adopting sequencing are required to elucidate the species in several taxonomic groups, as well as their distributions and vector status. Identifying the primary and secondary malaria vectors in such a region is critical for appropriate, targeted malaria control interventions and accurate monitoring of their effectiveness. Finally, the design and analysis used in this work represent a data set and methodologies that can be applied anywhere in southern Cambodia and northern Laos to make progress toward the objective of eliminating forest malaria.

## Supplementary Information

**Additional file 1: Table S1.** Full list of 442 *Anopheles* specimens which were classified by both molecular and morphological identification, with group ID, morphology species ID, molecular species ID based on ITS2, molecular species ID based on *COII*, geographical location, latitude, longitude and taxonomic classification.

**Additional file 2: Table S2.** Full list of the ITS2 and *COII* sequences of *Anopheles* spp. from original data, with sample ID and species. Sheet 1, elucidating a total of 412 ITS2 sequences covering 12 species, was used for the median-joining network, and 30 ITS2 sequences (haplotypes) were used for phylogenetic analysis. Sheet 2, elucidating a total of 391 *COII* sequences covering 13 species, was used for the median-joining network, and 199 *COII* sequences (haplotypes) were used for phylogenetic analysis.

**Additional file 3: Table S3.** Full list of the ITS2 and *COII* sequences of *Anopheles* spp. deposited in GenBank, with GenBank accession numbers and species. A total of 591 ITS2 sequences covering 12 species and 88 ITS2 sequences (haplotypes) used for phylogenetic analysis were elucidated in sheet 1, while a total of 873 *COII* sequences covering 13 species and 308 *COII* sequences (haplotypes) used for phylogenetic analysis were elucidated in sheet 2.

**Additional file 4: Figure S1.** Phylogenetic tree based on 1003 ITS2 sequences (118 haplotypes) from GenBank and our original data. Bootstrap values (1000 replicates) of maximum likelihood analyses are shown above/below the main lineages. Lineage designation is indicated on the right. Bars represent 2.0 substitutions per site based on ITS2.

**Additional file 5: Figure S2.** Phylogenetic tree based on 1264 *COII* sequences (507 haplotypes) from GenBank and our original data. Bootstrap values (1000 replicates) of maximum likelihood analyses are shown above/below the main lineages. Lineage designation is indicated on the right. Bars represent 5.0 substitutions per site based on *COII*.

**Additional file 6: Table S4.** Mean intra- and interspecific K2P distances of the ITS2 sequence (**a**) and *COII* sequence (**b**) in 12 *Anopheles* species. The numbers of intraspecific distances are shown in boldface for clarity. Numbers underlined indicate the highest intraspecific distance and the lowest interspecific distance. Abbreviations: n, no. of sequences; na, not applicable; *arg.*, *An. argyropus*; *nig.*, *An. nigerrimus*; *nit.*, *An. nitidus*; *ped.*, *An. peditaeniatus*; *sin.*, *An. sinensis*; *niv.*, *An. nivipes*; *tes.*, *An. tessellatus*; *dir.*, *An. dirus*; *mac.*, *An. maculatus*; *phi.*, *An. philippinensis*; *koc.*, *An. kochi*; *vag.*, *An. vagus*.

**Additional file 7: Figure S3.** Intra- and interspecific divergence determined using the Kimura 2-parameter distance; *y*-axis, genetic divergence; *x*-axis, species. (**a**) Genetic divergence of ITS2. The barcoding gap ranged from 0.042 to 0.192. (**b**) Genetic divergence of *COII*. The barcoding gap ranged from 0.033 to 0.047. *arg.*, *An. argyropus*; *nig.*, *An. nigerrimus*; *nit.*, *An. nitidus*; *ped.*, *An. peditaeniatus*; *sin.*, *An. sinensis*; *niv.*, *An. nivipes*; *tes.*, *An. tessellatus*; *dir.*, *An. dirus*; *mac.*, *An. maculatus*; *phi.*, *An. philippinensis*; *koc.*, *An. kochi*; *vag.*, *An. vagus*.

**Additional file 8: Figure S4.** ITS2 and *COII* sequence divergence. Each dot represents a species, with interspecific distance on the *y*-axis and intraspecific distance on the *x*-axis. The minimum interspecific (intergroup) divergence is plotted against the maximum intraspecific divergence. Red and green dots indicate the ITS2 and *COII* sequence divergence of 12 species including *An. argyropus*, *An. nigerrimus*, *An. nitidus*, *An. peditaeniatus*, *An. sinensis*, *An. nivipes*, *An. tessellatus*, *An. dirus*, *An. maculatus*, *An. philippinensis*, *An. kochi* and *An. vagus*.

**Additional file 9: Figure S5.** Phylogenetic tree based on 18 ITS2 sequences (**a**) and 15 *COII* sequences (**b**) of *Anopheles tessellatus* from GenBank and our original data. Bootstrap values (1000 replicates) of maximum likelihood analyses are shown above/below the main lineages. Lineage designation is indicated on the right. Bars represent 2.0 substitutions per site based on ITS2 and 0.6 substitutions per site based on *COII*.

## Data Availability

Data supporting the conclusions of this article are included within the article and its additional files. The data sets generated and/or analyzed during the current study are available in GenBank (http://www.ncbi.nlm.nih.gov/). The raw data sets used and/or analyzed during this study are available from the corresponding author upon reasonable request.

## Abbreviations

ITS2: Internal transcribed spacer 2
*COII*: Cytochrome c oxidase subunit 2
ML: Maximum likelihood
K2P: Kimura 2-parameter
GMS: Greater Mekong Subregion
ITNs: Insecticide-treated nets
RMT: Residual malaria transmission
