# Supplementary figures and images for: Morphological and molecular identification reveals a high diversity of Anopheles species in the forest region of the Cambodia–Laos border
Source: Parasit Vectors. 2022 Mar 18;15:94. doi: 10.1186/s13071-022-05167-0 (PMC8933986; doi:10.1186/s13071-022-05167-0)

Neomyzomyia Series

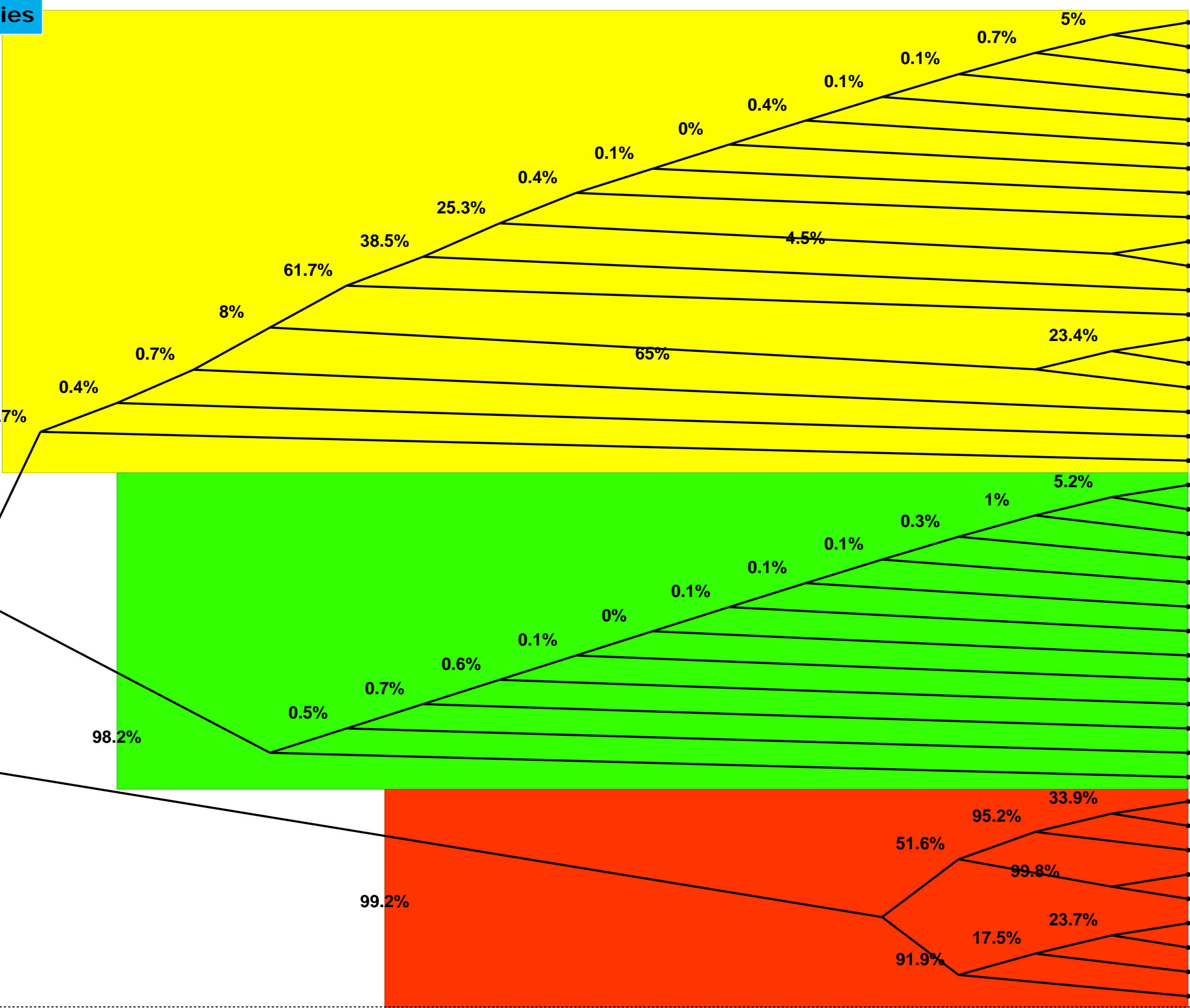

Myzorhynchus Series

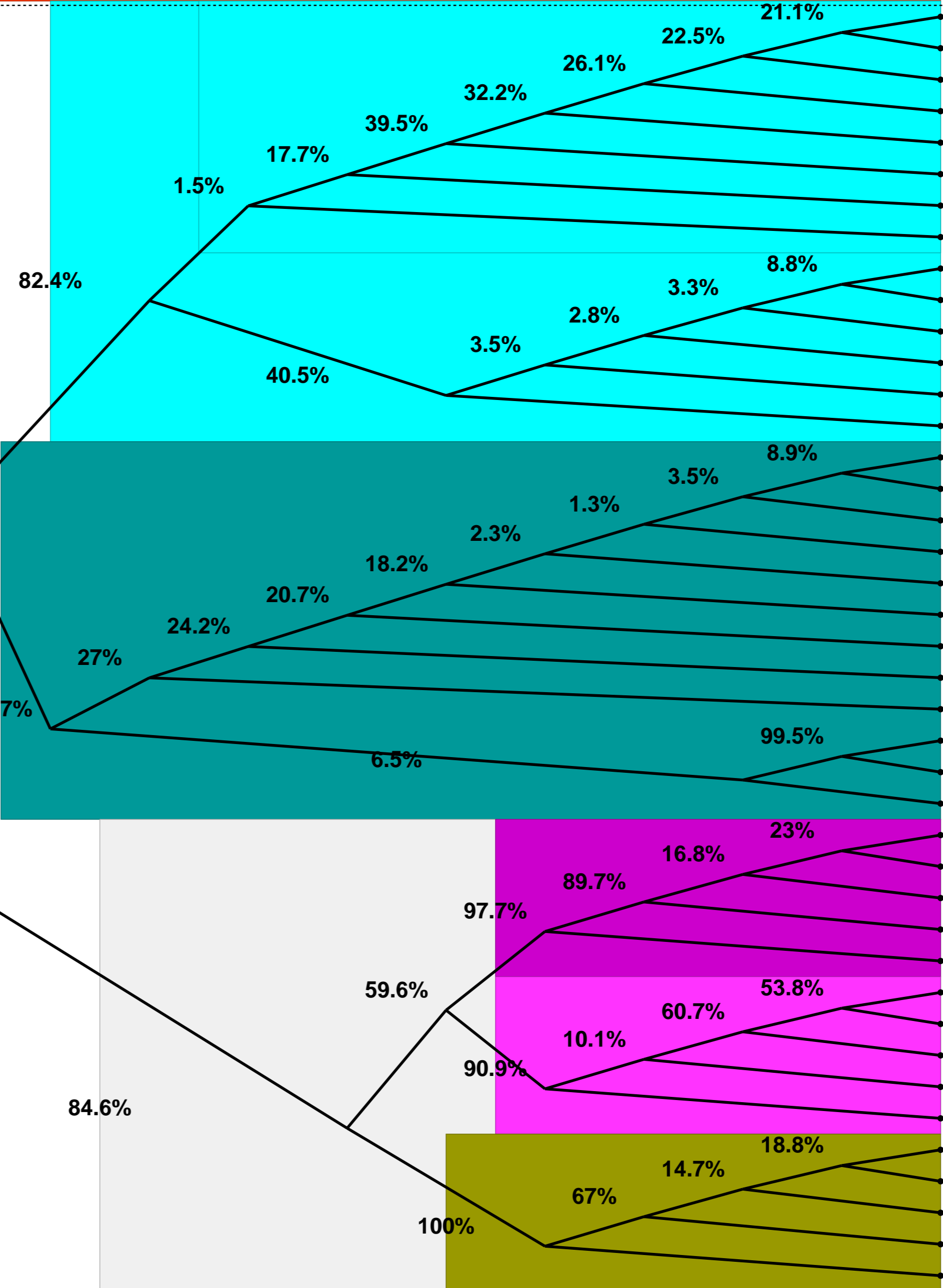

Neocellia Series

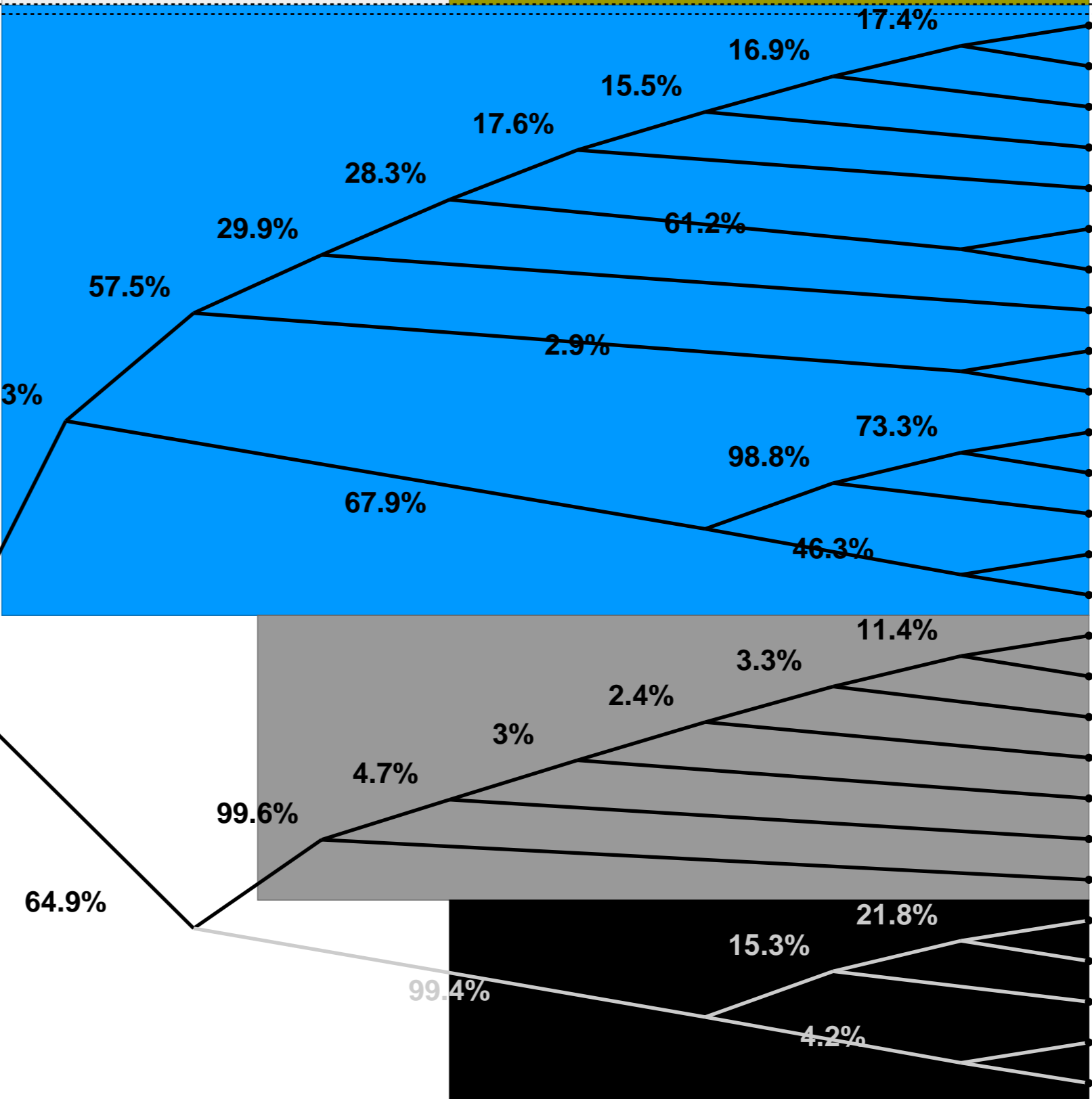

Pyretophorus Series

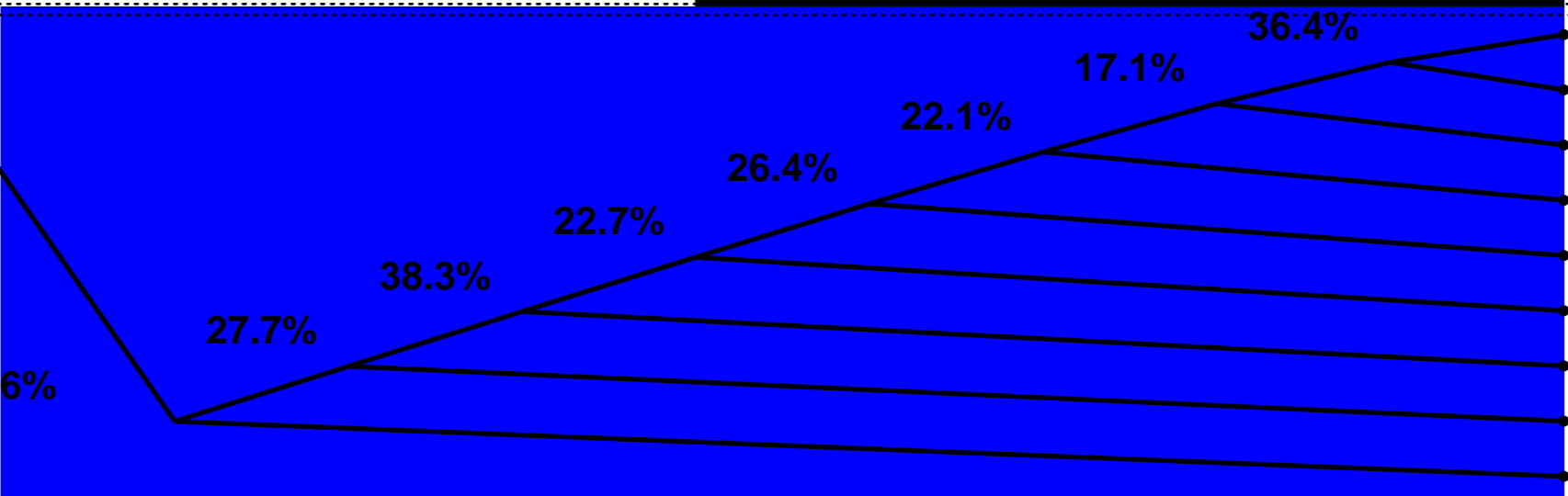

Supplement: Supplementary file 4 — Additional file 4: Figure S1. Phylogenetic tree based on 1003 ITS2 sequences (118 haplotypes) from GenBank and our original data. Bootstrap values (1000 replicates) of maximum likelihood analyses are shown above/below the main lineages. Lineage designation is indicated on the right. Bars represent 2.0 substitutions per site based on ITS2. [file 13071_2022_5167_MOESM4_ESM.pdf]

**a**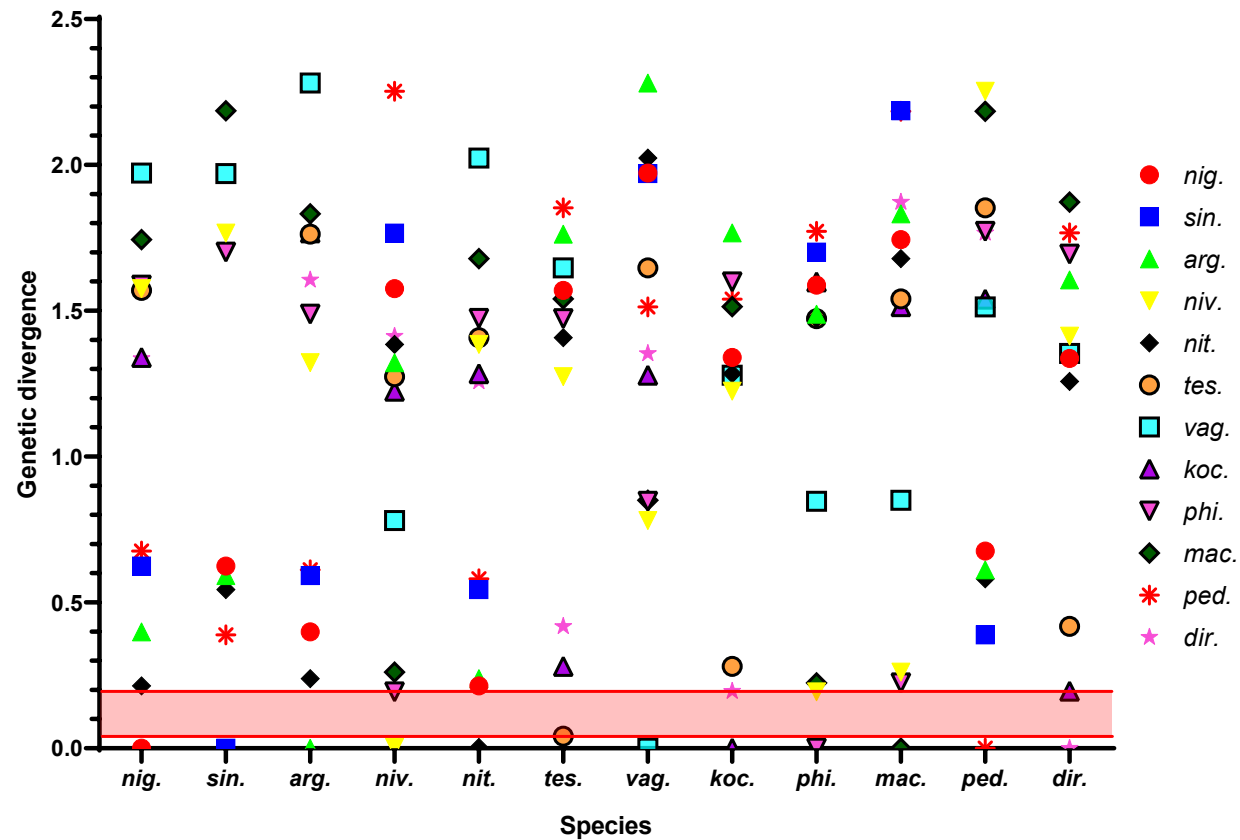**b**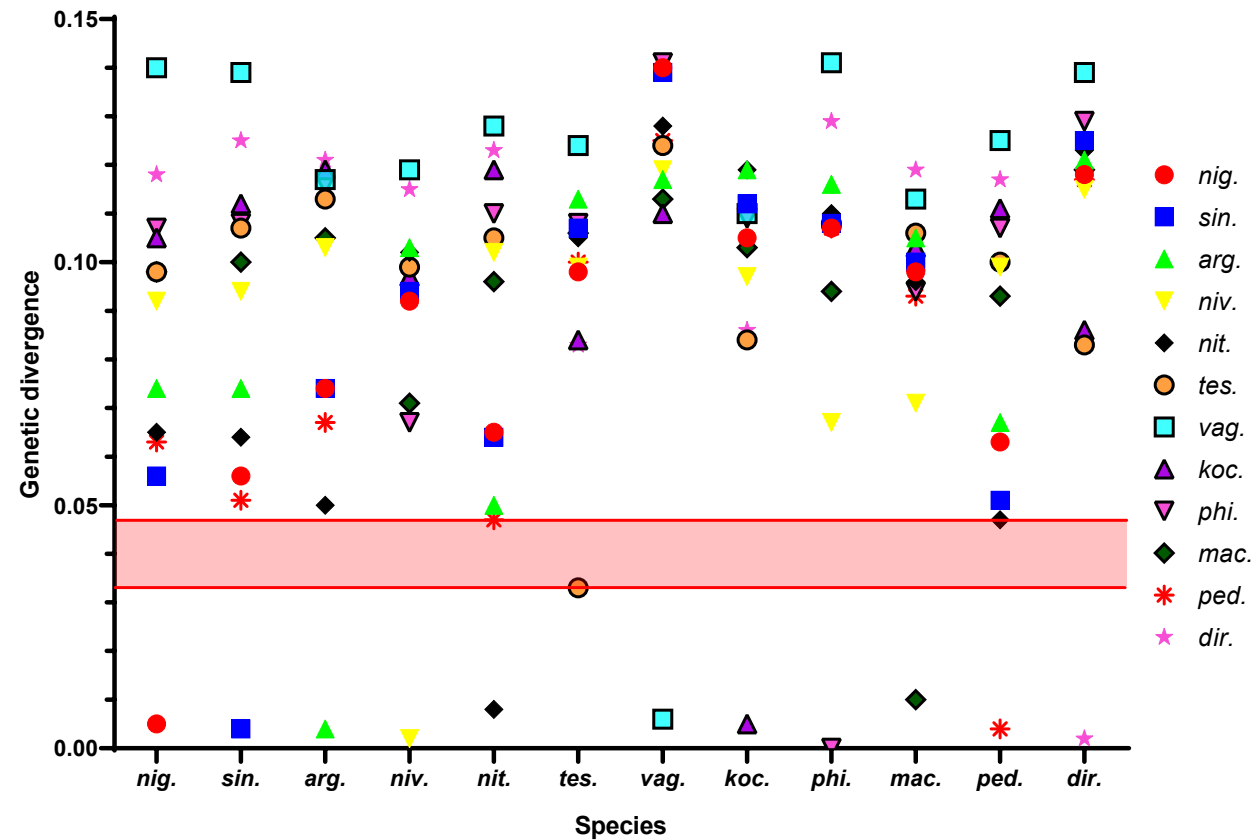

Supplement: Supplementary file 7 — Additional file 7: Figure S3. Intra- and interspecific divergence determined using the Kimura 2-parameter distance; y-axis, genetic divergence; x-axis, species. (a) Genetic divergence of ITS2. The barcoding gap ranged from 0.042 to 0.192. (b) Genetic divergence of COII. The barcoding gap ranged from 0.033 to 0.047. arg., An. argyropus; nig., An. nigerrimus; nit., An. nitidus; ped., An. peditaeniatus; sin., An. sinensis; niv., An. nivipes; tes., An. tessellatus; dir., An. dirus; mac., An. maculatus; phi., An. philippinensis; koc., An. kochi; vag., An. vagus. [file 13071_2022_5167_MOESM7_ESM.pdf]

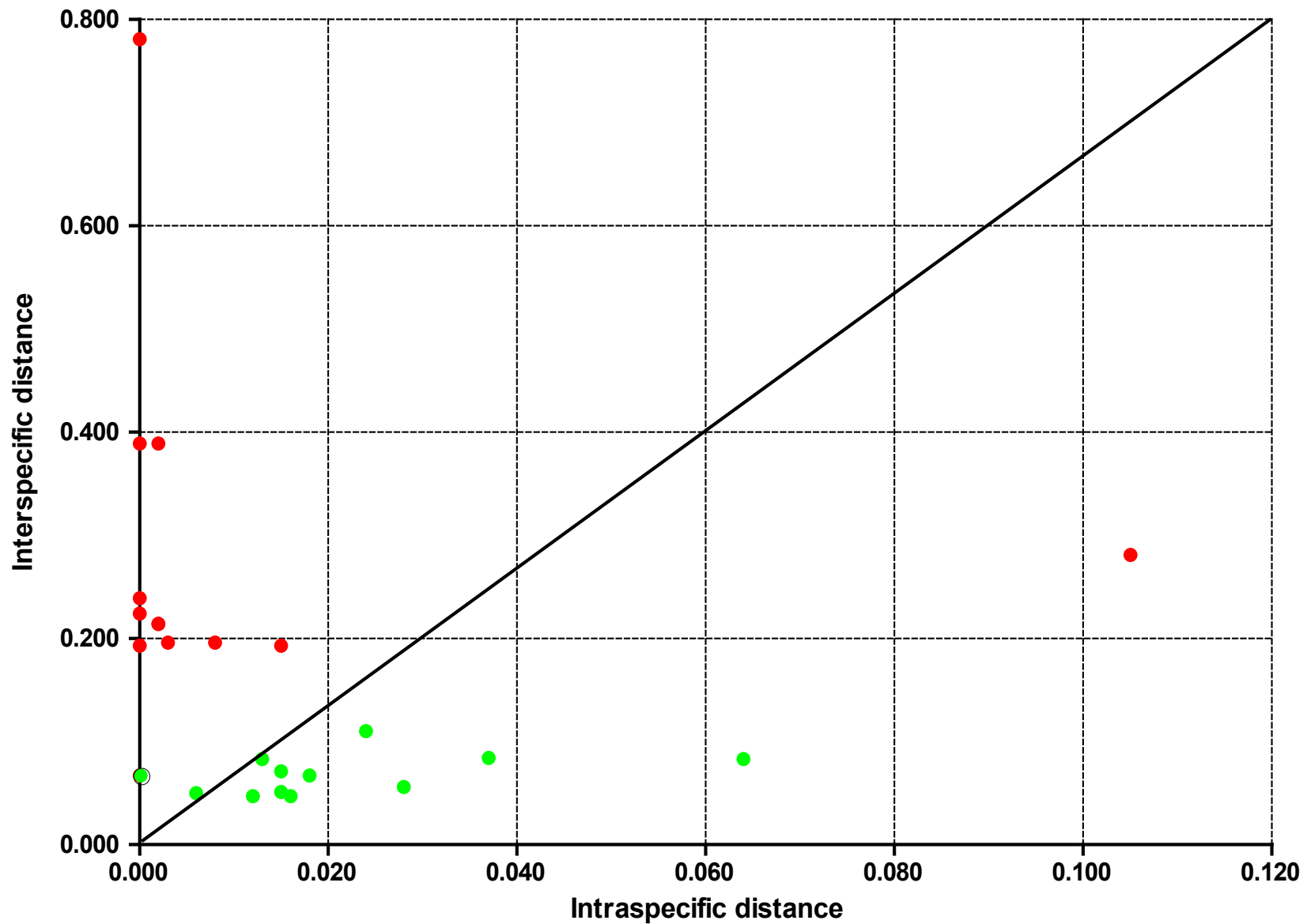

Supplement: Supplementary file 8 — Additional file 8: Figure S4. ITS2 and COII sequence divergence. Each dot represents a species, with interspecific distance on the y-axis and intraspecific distance on the x-axis. The minimum interspecific (intergroup) divergence is plotted against the maximum intraspecific divergence. Red and green dots indicate the ITS2 and COII sequence divergence of 12 species including An. argyropus, An. nigerrimus, An. nitidus, An. peditaeniatus, An. sinensis, An. nivipes, An. tessellatus, An. dirus, An. maculatus, An. philippinensis, An. kochi and An. vagus. [file 13071_2022_5167_MOESM8_ESM.pdf]

**a**

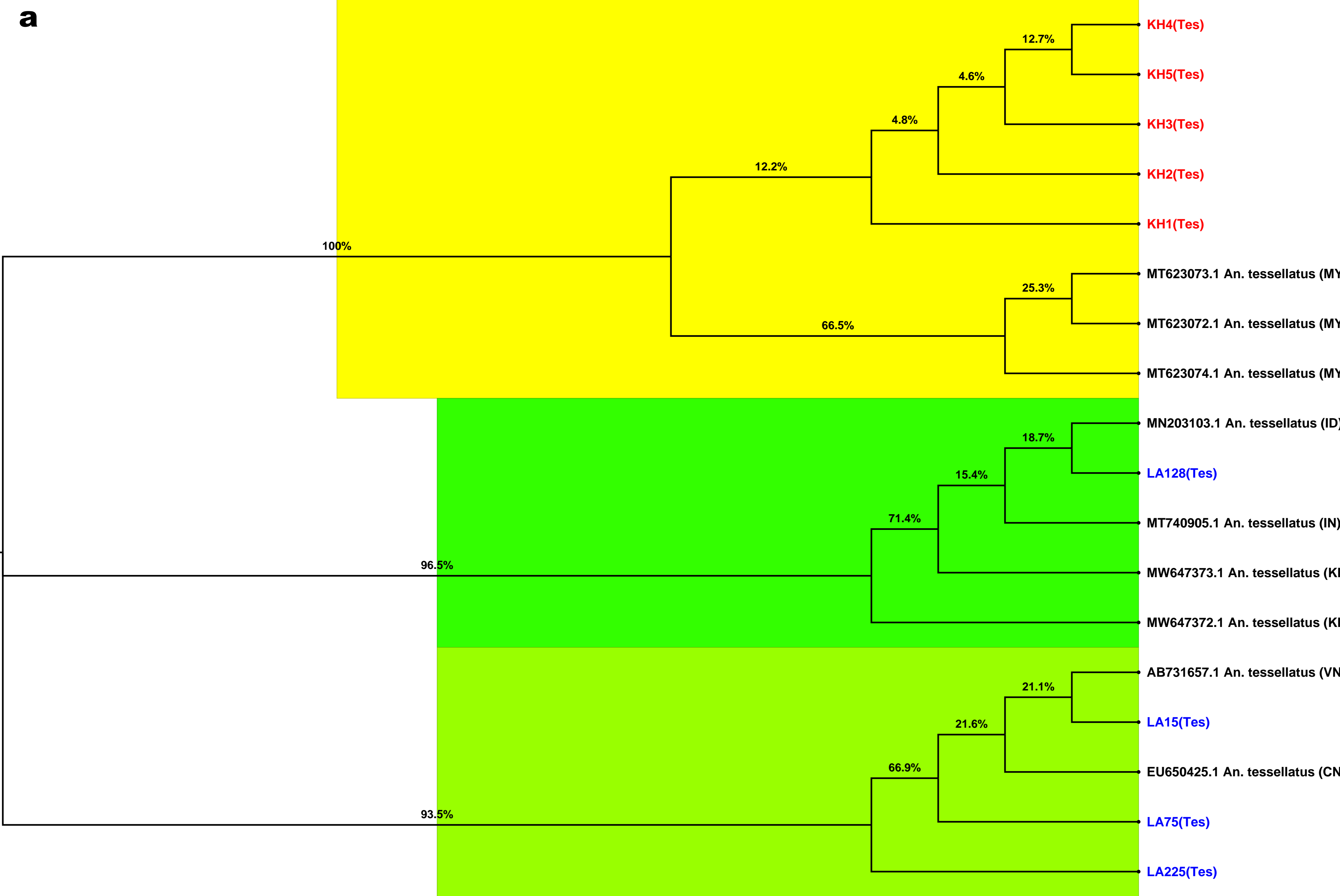

**b**

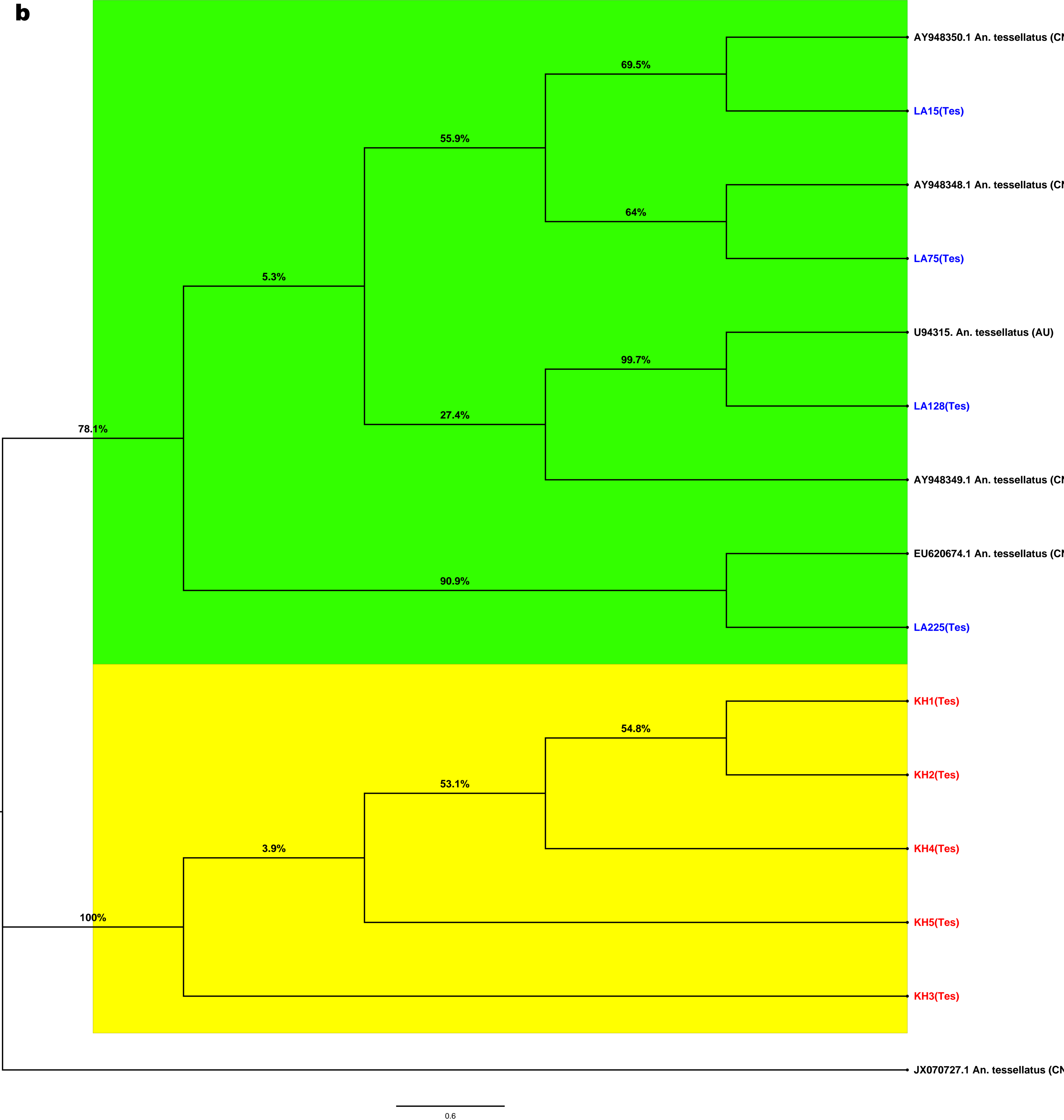

Supplement: Supplementary file 9 — Additional file 9: Figure S5. Phylogenetic tree based on 18 ITS2 sequences (a) and 15 COII sequences (b) of Anopheles tessellatus from GenBank and our original data. Bootstrap values (1000 replicates) of maximum likelihood analyses are shown above/below the main lineages. Lineage designation is indicated on the right. Bars represent 2.0 substitutions per site based on ITS2 and 0.6 substitutions per site based on COII. [file 13071_2022_5167_MOESM9_ESM.pdf]
