# Supplementary material for: Morphological and molecular identification reveals a high diversity of Anopheles species in the forest region of the Cambodia–Laos border
Source: Parasit Vectors. 2022 Mar 18;15:94. doi: 10.1186/s13071-022-05167-0 (PMC8933986; doi:10.1186/s13071-022-05167-0)
Supplement: Supplementary file 6 — Additional file 6: Table S4. Mean intra- and interspecific K2P distances of the ITS2 sequence (a) and COII sequence (b) in 12 Anopheles species. The numbers of intraspecific distances are shown in boldface for clarity. Numbers underlined indicate the highest intraspecific distance and the lowest interspecific distance. Abbreviations: n, no. of sequences; na, not applicable; arg., An. argyropus; nig., An. nigerrimus; nit., An. nitidus; ped., An. peditaeniatus; sin., An. sinensis; niv., An. nivipes; tes., An. tessellatus; dir., An. dirus; mac., An. maculatus; phi., An. philippinensis; koc., An. kochi; vag., An. vagus. [file 13071_2022_5167_MOESM6_ESM.docx]

**Table S4a. Mean intra- and interspecific K2P distances of the ITS2 sequence in 12 *Anopheles* species.**

| Species | n | *nig.* | *sin.* | *arg.* | *niv.* | *nit.* | *tes.* | *vag.* | *koc.* | *phi.* | *mac.* | *ped.* | *dir.* |
| --- | --- | --- | --- | --- | --- | --- | --- | --- | --- | --- | --- | --- | --- |
| *nig.* | 14 | **0.000** |  |  |  |  |  |  |  |  |  |  |  |
| *sin.* | 4 | 0.625 | **0.000** |  |  |  |  |  |  |  |  |  |  |
| *arg.* | 8 | 0.399 | 0.592 | **0.000** |  |  |  |  |  |  |  |  |  |
| *niv.* | 53 | 1.576 | 1.766 | 1.322 | **0.003** |  |  |  |  |  |  |  |  |
| *nit.* | 3 | 0.214 | 0.544 | 0.239 | 1.385 | **0.003** |  |  |  |  |  |  |  |
| *tes.* | 9 | 1.570 | na | 1.763 | 1.274 | 1.408 | **0.042** |  |  |  |  |  |  |
| *vag.* | 11 | 1.972 | 1.970 | 2.281 | 0.781 | 2.024 | 1.647 | **0.000** |  |  |  |  |  |
| *koc.* | 211 | 1.340 | na | 1.767 | 1.224 | 1.284 | 0.281 | 1.279 | **0.000** |  |  |  |  |
| *phi.* | 2 | 1.588 | 1.701 | 1.488 | 0.193 | 1.474 | 1.473 | 0.847 | 1.599 | **0.000** |  |  |  |
| *mac.* | 7 | 1.744 | 2.186 | 1.832 | 0.261 | 1.679 | 1.541 | 0.850 | 1.514 | 0.224 | **0.000** |  |  |
| *ped.* | 18 | 0.676 | 0.389 | 0.612 | 2.253 | 0.581 | 1.853 | 1.513 | 1.540 | 1.772 | 2.184 | **0.000** |  |
| *dir.* | 21 | 1.337 | na | 1.606 | 1.413 | 1.258 | 0.418 | 1.354 | 0.196 | 1.694 | 1.873 | 1.767 | **0.000** |

**Table S4b. Mean intra- and interspecific K2P distances of the COII sequence in 12 *Anopheles* species.**

| Species | n | *nig.* | *sin.* | *arg.* | *niv.* | *nit.* | *tes.* | *vag.* | *koc.* | *phi.* | *mac.* | *ped.* | *dir.* |
| --- | --- | --- | --- | --- | --- | --- | --- | --- | --- | --- | --- | --- | --- |
| *nig.* | 14 | **0.005** |  |  |  |  |  |  |  |  |  |  |  |
| *sin.* | 4 | 0.056 | **0.004** |  |  |  |  |  |  |  |  |  |  |
| *arg.* | 8 | 0.074 | 0.074 | **0.004** |  |  |  |  |  |  |  |  |  |
| *niv.* | 53 | 0.092 | 0.094 | 0.103 | **0.002** |  |  |  |  |  |  |  |  |
| *nit.* | 3 | 0.065 | 0.064 | 0.050 | 0.102 | **0.008** |  |  |  |  |  |  |  |
| *tes.* | 9 | 0.098 | 0.107 | 0.113 | 0.099 | 0.105 | **0.033** |  |  |  |  |  |  |
| *vag.* | 11 | 0.140 | 0.139 | 0.117 | 0.119 | 0.128 | 0.124 | **0.006** |  |  |  |  |  |
| *koc.* | 211 | 0.105 | 0.112 | 0.119 | 0.097 | 0.119 | 0.084 | 0.110 | **0.005** |  |  |  |  |
| *phi.* | 2 | 0.107 | 0.108 | 0.116 | 0.067 | 0.110 | 0.108 | 0.141 | 0.109 | **0.000** |  |  |  |
| *mac.* | 7 | 0.098 | 0.100 | 0.105 | 0.071 | 0.096 | 0.106 | 0.113 | 0.103 | 0.094 | **0.010** |  |  |
| *ped.* | 18 | 0.063 | 0.051 | 0.067 | 0.099 | 0.047 | 0.100 | 0.125 | 0.111 | 0.107 | 0.093 | **0.004** |  |
| *dir.* | 21 | 0.118 | 0.125 | 0.121 | 0.115 | 0.123 | 0.083 | 0.139 | 0.086 | 0.129 | 0.119 | 0.117 | **0.002** |

The numbers of intraspecific distances are shown in boldface for clarity. Numbers underlined indicate the highest intraspecific distance and the lowest interspecific distance. Abbreviations: n, no.of sequences; na, not applicable; *arg.*, *An. argyropus*; *nig.*, *An. nigerrimus*; *nit.*, *An. nitidus*; *ped.*, *An. peditaeniatus*; *sin.*, *An. sinensis*; *niv.*, *An. nivipes*; *tes.*, *An. tessellatus*; *dir.*, *An. dirus*; *mac.*, *An. maculatus*; *phi.*, *An. philippinensis*; *koc.*, *An. kochi*; *vag.*, *An. vagus*.
