# Supplementary material for: Morphological and molecular identification reveals a high diversity of Anopheles species in the forest region of the Cambodia–Laos border
Source: Parasit Vectors. 2022 Mar 18;15:94. doi: 10.1186/s13071-022-05167-0 (PMC8933986; doi:10.1186/s13071-022-05167-0)
Supplement: Supplementary file 5 — Additional file 5: Figure S2. Phylogenetic tree based on 1264 COII sequences (507 haplotypes) from GenBank and our original data. Bootstrap values (1000 replicates) of maximum likelihood analyses are shown above/below the main lineages. Lineage designation is indicated on the right. Bars represent 5.0 substitutions per site based on COII. [file 13071_2022_5167_MOESM5_ESM.pdf]

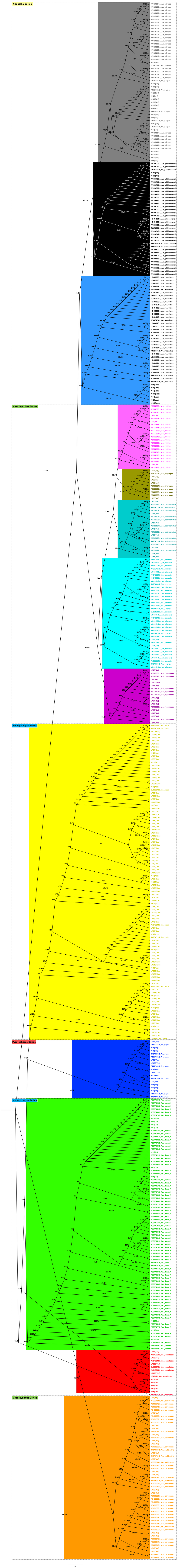
Neocellia Series
Myzorhynchus Series
Neomyzomyia Series
Pyretophorus Series
Neomyzomyia Series
Myzorhynchus Series
